# Aorto-ventricular tunnel with three orifices: a unique case report diagnosed by transthoracic echocardiography

**DOI:** 10.1186/s12947-023-00303-x

**Published:** 2023-04-01

**Authors:** Canying Yang, Juesheng Yang, Xiaoning Huang, Jiwei Wang

**Affiliations:** 1 Department of Ultrasound, The Second Affiliated Hospital of Nanchang University, Nanchang, 330006 Jiangxi China; 2 Department of Cardiovascular Surgery, The Second Affiliated Hospital of Nanchang University, Nanchang, 330006 Jiangxi China; 3 Department of Imaging Center, The Second Affiliated Hospital of Nanchang University, Nanchang, 330006 Jiangxi China

**Keywords:** Aorto-ventricular tunnel, Aorto-left ventricular tunnel, Aorto-right ventricular tunnel, Aorto-biventricular tunnel, Transthoracic echocardiography

## Abstract

**Background:**

Aorto-ventricular tunnel (AVT) is an abnormal communication channel between the ascending aorta and the ventricle. It commonly has two orifices, i.e., one aortic opening and one ventricular opening. In this study, we present a unique case of AVT with three orifices: one aortic opening, one LV opening, and one RV opening.

**Case presentation:**

A 64-year-old male presented with chest discomfort and dyspnea on exertion lasting the past six months. Physical examination revealed a grade 4/6 continuous biphasic murmur along the left sternal edge and a grade 3/6 systolic murmur at the apex. Transthoracic echocardiography (TTE) demonstrated: (1) an AVT with three orifices, i.e., one aortic opening, one LV opening, and one RV opening. The LV and RV openings were located in the left and right ventricular outflow tracts, respectively. (2) The aortic valve (AV) was calcified with a small aneurysm at the non-coronary cusp. (3)The mitral valve (MV) chordal rupture of the P2 and P3 segments was observed in the posterior leaflet with severe eccentric regurgitation. Subsequent coronary computed tomography angiography (CTA) further confirmed the diagnosis of AVT with three openings, and clarified the coronary arteries normally arose from the aortic sinuses. The patient was then referred for surgical treatment, consisting of closure of three AVT orifices, AV replacement, and MV replacement. Six months following surgery, the patient was asymptomatic. TTE demonstrated normal mechanic AV and MV function, and there was no residual shunt among the ascending aorta, LV and RV.

**Conclusions:**

It is the first case to report an AVT with three orifices. This paper described the entire process from diagnosis to treatment of this unique case, thus providing some novel insights into AVT.

**Supplementary Information:**

The online version contains supplementary material available at 10.1186/s12947-023-00303-x.

## Background

Aorto-ventricular tunnel (AVT) is an abnormal communication channel between the ascending aorta and the ventricle. It commonly has two orifices, i.e., one aortic opening and one ventricular opening. The aortic opening usually lies above the right coronary sinus and rarely above the left and non-coronary sinus, while the ventricular opening can be in the left ventricle (LV) or right ventricle (RV) but is mainly located in the left/right ventricular outflow tract [1].

**Fig. 1:**
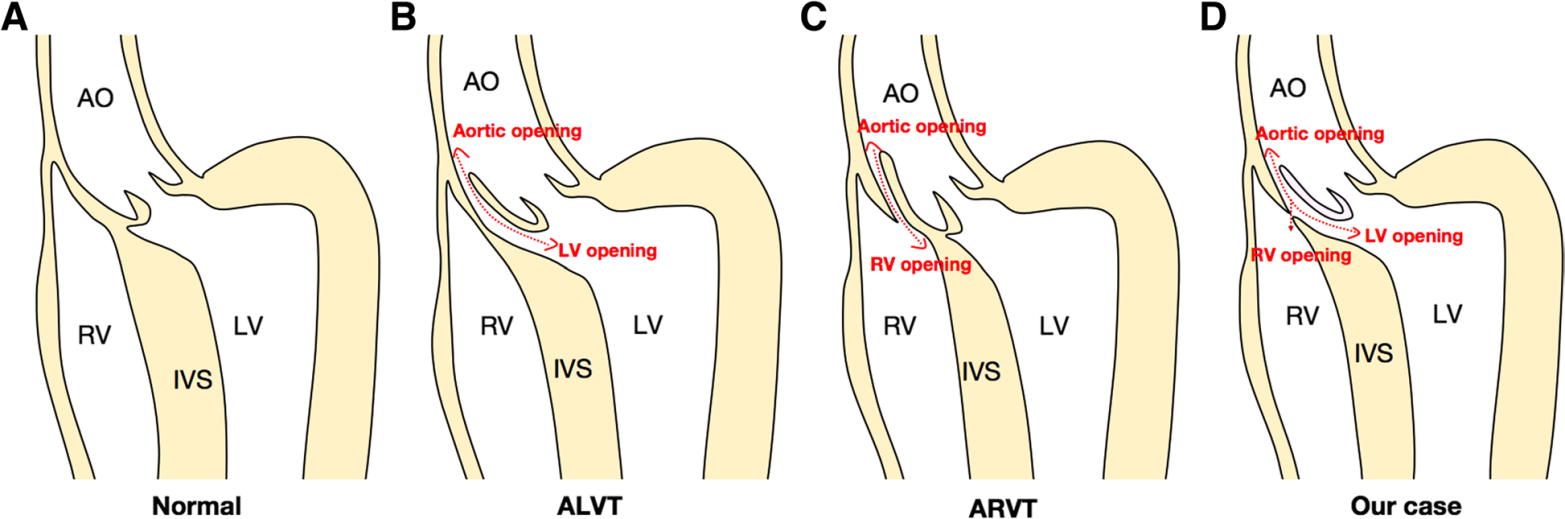
Schematic diagram of the AVT. **A** Normal structure without AVT. **B** ALVT with two orifices. **C** ARVT with two orifices. **D** AVT with three orifices in our case

AVT is a rare congenital heart defect first described in 1963 by Levy et al. [2]. Since then, approximately 250 cases of the aorto-left ventricular tunnel (ALVT) and 30 cases of aorto-right ventricular tunnel (ARVT) have been reported in the English literature. In this paper, we presented a unique case of AVT with three orifices, i.e., one aortic opening, one LV opening, and one RV opening (Fig. 1). To the best of our knowledge, no such cases have been reported in the English literature so far.

## Case presentation

**Fig. 2:**
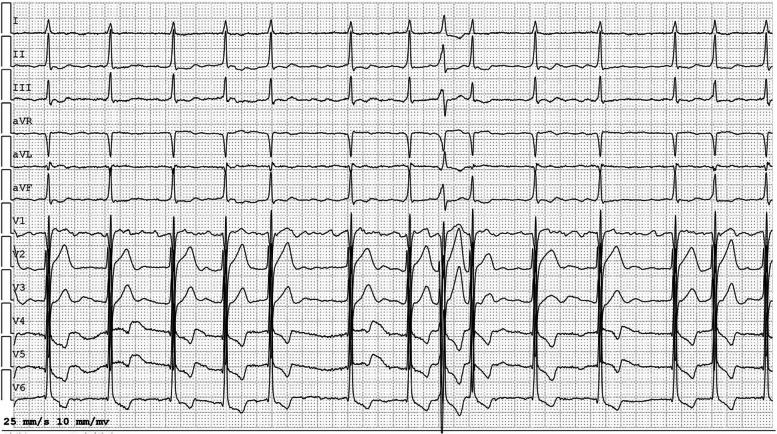
ECG image. The electrocardiogram showed atrial fibrillation, ventricular premature, left ventricular hypertrophy, and ST-T wave abnormalities

**Fig. 3:**
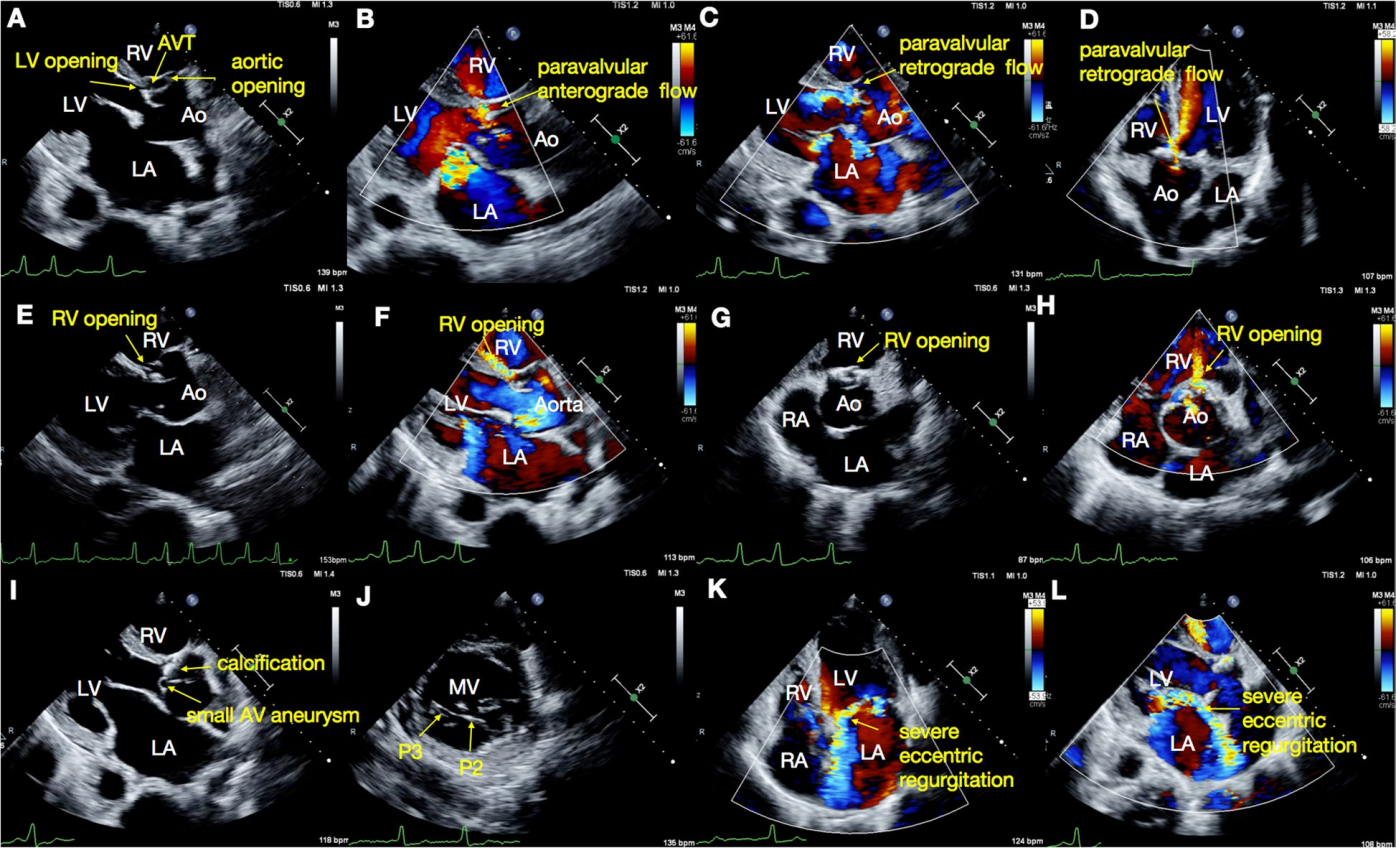
TTE images. **A** An AVT was noted, and its aortic and LV opening was observed in the parasternal long-axis view. **B**-**C** Color Doppler flow imaging (CDFI) revealed the anterograde systolic and retrograde diastolic flow between the aorta and the LV via the AVT in the parasternal long-axis view. **D** The retrograde diastolic flow via the AVT was also shown in the apical five-chamber view. **E**–**F** With a slight tilt of the probe, an RV opening was observed from the AVT in the modified parasternal long-axis view, and CDFI displayed a flow jet from the AVT to the RV. **G**-**H** The RV opening was also confirmed in the short-axis view. **I** The AV was calcified with a small aneurysm of the non-coronary cusp. **J**-**L** The MV chordal rupture of the P2 and P3 segments was observed in the posterior leaflet with severe eccentric regurgitation

A 64-year-old male presented with chest discomfort and dyspnea on exertion lasting the past six months. His past medical history was unremarkable. Physical examination revealed a grade 4/6 continuous biphasic murmur along the left sternal edge and a grade 3/6 systolic murmur at the apex. His blood pressure was 124/66 mmHg, and his heart rate was 86 beats/min. The patient had no Marfan traits, and the electrocardiogram (ECG) showed atrial fibrillation, ventricular premature, left ventricular hypertrophy, and ST-T wave abnormalities (Fig. 2). Transthoracic echocardiography (TTE) demonstrated: (1) An enlarged heart with a reduced left ventricular ejection fraction of 42%. (2) An AVT was noted, and its aortic and LV opening was observed in the parasternal long-axis view (Fig. 3A, Video 1). Color Doppler flow imaging (CDFI) revealed the anterograde systolic and retrograde diastolic flow between the aorta and the LV via the AVT in the parasternal long-axis view (Fig. 3B-C, Video 2). The retrograde diastolic flow via the AVT was also shown in the apical five-chamber view (Fig. 3D, Video 3). (3) With a slight tilt of the probe, an RV opening was observed from the AVT in the modified parasternal long-axis view, and CDFI displayed a flow jet from the AVT to the RV (Fig. 3E-F, Videos 4 and 5). The RV opening was also confirmed in the short-axis view (Fig. 3G-H, Videos 6 and 7), and a biphasic spectrum was detected at the RV opening with a peak velocity of 489 cm/s. (4) The aortic valve (AV) was calcified with a small aneurysm of the non-coronary cusp (Fig. 3I). (5) The mitral valve (MV) chordal rupture of the P2 and P3 segments was observed in the posterior leaflet with severe eccentric regurgitation (Fig. 3J-L). Detailed cardiac measurement parameters are shown in Table 1.

**Table 1:**
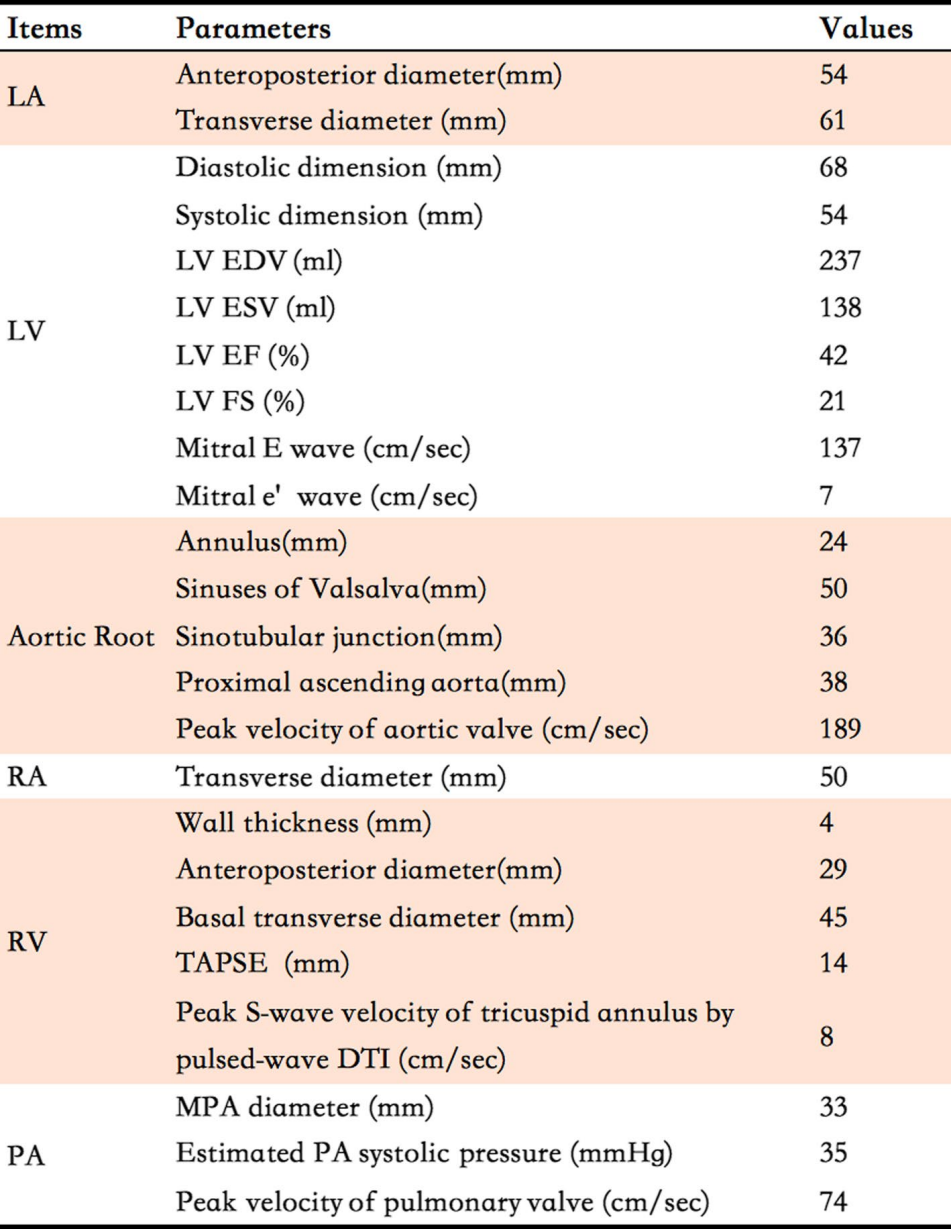
Cardiac parameters measured by TTE

* LA* Left atrium, *LV* Left ventricle, *RV* Right ventricle, *RA* Right atrium, *PA* Pulmonary artery, *EDV* End-diastolic volume, *ESV* End-systolic volume, *EF* Ejection fraction, *FS* Fractional shortening, *TAPSE* The tricuspid annular plane systolic excursion, *DTI* Doppler tissue imaging, *MPA* Main pulmonary artery

**Fig. 4:**
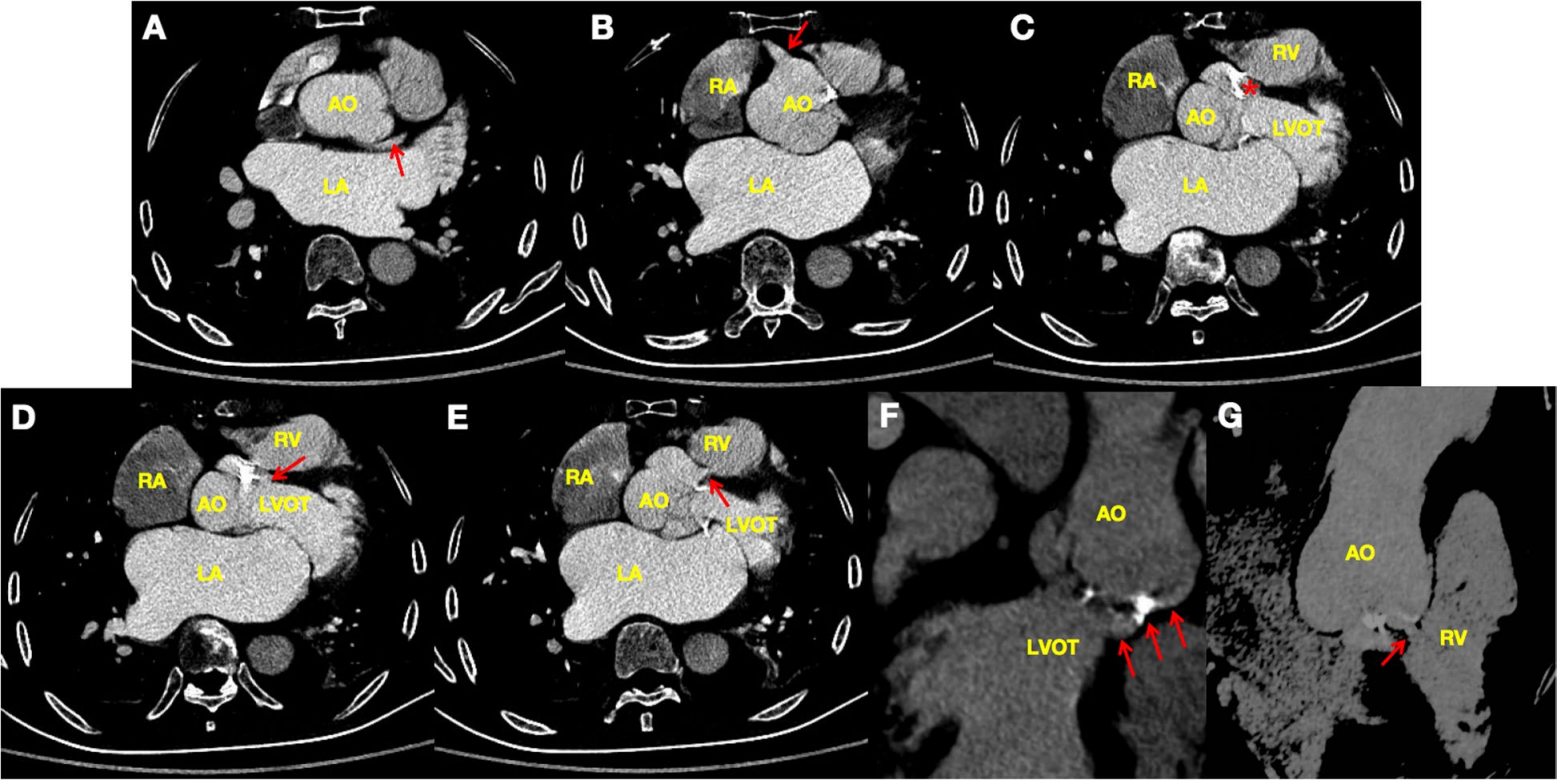
CTA images. **A** Origin of the LCA (arrow) from the left coronary sinus. **B** Origin of the RCA (arrow) from the right coronary sinus. **C** An AVT (asterisk) is anteriorly and laterally located to the right coronary sinus. **D** The LV opening (arrow) of the AVT was in the left ventricular outflow tract. **E** The RV opening (arrow) of the AVT was in the right ventricular outflow tract. **F** The AVT (arrow) was shown by 3D reconstruction. **G** The RV opening (arrow) was shown by 3D reconstruction

**Fig. 5:**
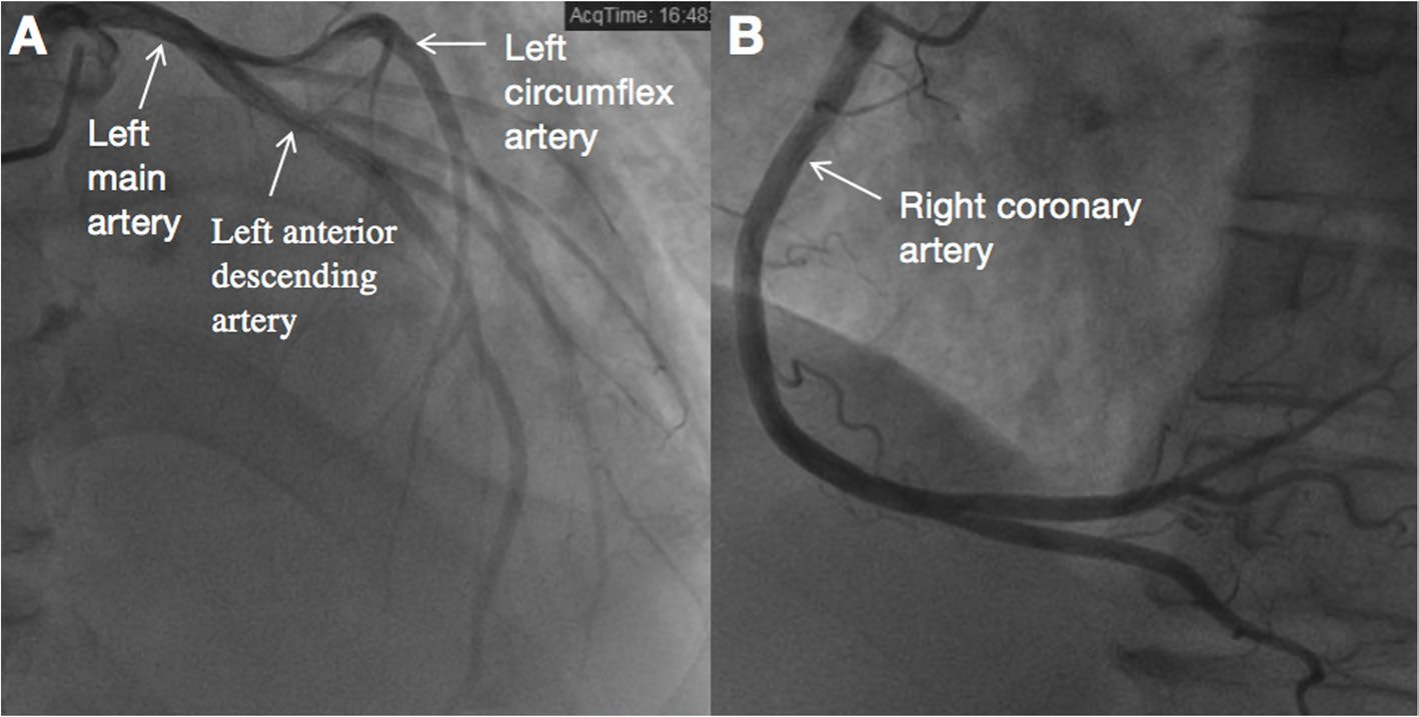
CAG images. **A** The left coronary artery normally arose from the left aortic sinus with grade 3 TIMI blood flow. **B** The right coronary artery normally arose from the right aortic sinus with grade 3 TIMI blood flow

Computed tomography angiography (CTA) revealed: (1) both coronary arteries normally arose from the aortic sinuses (Fig. 4A-B); (2) An AVT is anteriorly and laterally located to the right coronary sinus (Fig. 4C); (3) The LV opening was in the LV outflow tract (Fig. 4D); (4) The RV opening was in the RV outflow tract (Fig. 4E). With 3D reconstruction, the AVT and its RV opening were shown intuitively (Fig. 4F-G). Coronary artery angiography (CAG) further confirmed that coronary arteries originated normally arose from the aortic sinuses and revealed complete perfusion of grade 3 TIMI blood flow (Fig. 5A-B).

Laboratory analysis showed a mildly elevated N-terminal pro-brain natriuretic peptide (NT-pro-BNP) of 641 pg. Other laboratory tests were within normal reference ranges, such as complete blood count, serum electrolytes, coagulation function, hepatic function, renal function, autoimmune markers, and inflammatory markers. The main parameters of the laboratory test could be obtained from the supplementary Table S1.

**Fig. 6:**
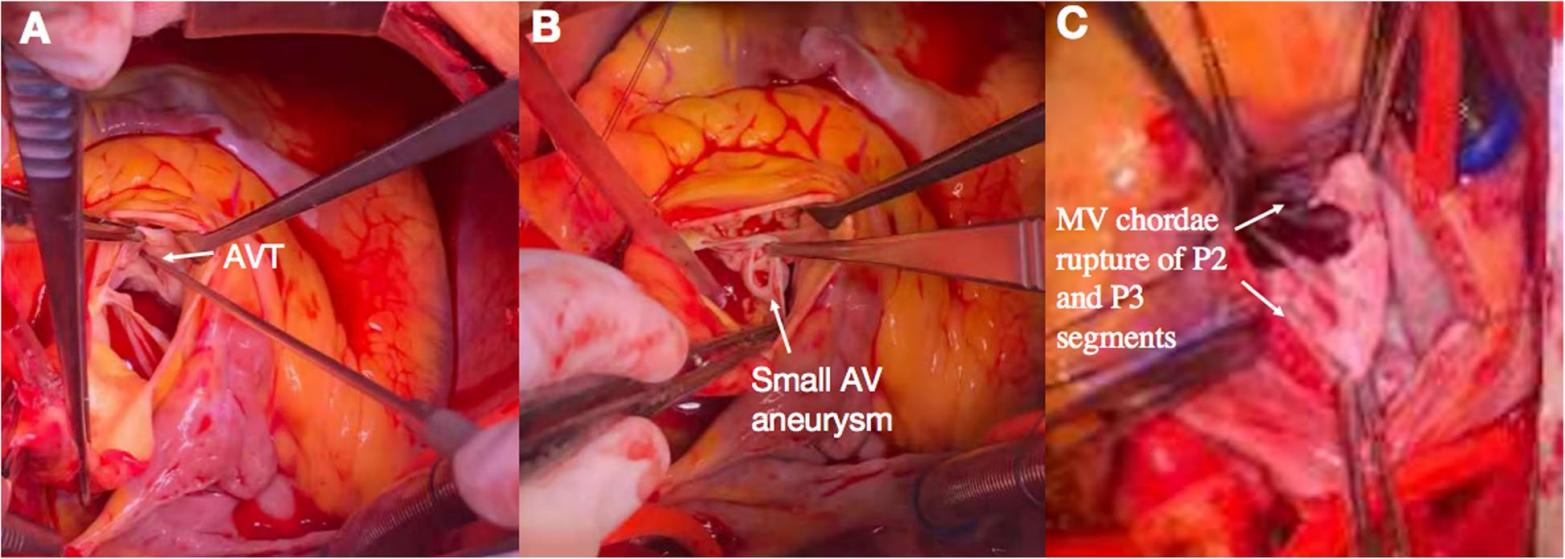
Intraoperative images. **A** The aortic opening was above the right coronary sinus, the LV opening was in the LV outflow tract, and the RV opening was in the RV outflow tract. The nerve retractor could detect the LV and RV openings from the aortic opening. **B** The AV was tricuspid with areas of calcification and a small aneurysm at the non-coronary cusp. **C** The MV chordal rupture of the P2 and P3 segments was observed in the posterior leaflet

The patient subsequently underwent surgery. At operation, the AVT with three orifices was confirmed. The aortic opening was above the right coronary sinus, the LV opening was in the LV outflow tract, and the RV opening was in the RV outflow tract. The nerve retractor could detect the LV and RV openings from the aortic opening (Fig. 6A). The AV was tricuspid with areas of calcification and a small aneurysm at the non-coronary cusp (Fig. 6B). There was evidence of chordal rupture of the P2 and P3 segments of the MV posterior leaflet (Fig. 6C). AVT was repaired with a 5–0 Prolene suture with pledgets to close the aortic opening and 4–0 Prolene with a pericardial patch to close the LV and RV opening. Due to calcification and dysfunction, the AV was replaced with a bileaflet mechanical valve (23 mm, St. Jude regent, USA). Considering that the patient was elderly with a significantly enlarged LV, the MV was replaced with a bileaflet mechanical valve (27 mm, Sorin Biomedica, Italy) instead of mitral repair.

Microscopic pathologic examination of the excised AV and MV revealed fibrosis, hyalinization, and areas of necrosis and calcification. The postoperative recovery was uneventful. Six months later, the patient was asymptomatic. TTE demonstrated normal mechanic AV and MV function, and there was no residual shunt among the ascending aorta, LV and RV. The systolic function of both ventricles was normal.

## Discussion and conclusions

AVT is a rare congenital cardiac malformation characterized by abnormal communication between the ascending aorta and the ventricle. The incidence of ALVT ranges between 0.001% and 0.1% of all postnatal congenital heart defects but is much higher in prenatal heart specimens, reaching 0.5% [3–5]. The ARVT is rarer, with about one-eighth of the AVT communicating with the RV [6]. AVT commonly has two orifices, i.e., aortic opening and ventricular opening. There are no previous reports on AVT with three orifices. This paper presented a peculiar and unique case of AVT with one aortic opening, one LV opening, and one RV opening. The patient had never taken an echocardiographic assessment, so it is hard to say whether the RV opening is congenital or acquired. If it is congenital, it could be termed an “aorto-biventricular tunnel.” However, it is possible that the RV opening was spontaneous rupture due to the shear stress caused by the long-term shunt of the ALVT, and if this was the case, such a condition could be termed “ALVT with a breach to the RV.”

AVT often presents during infancy or early childhood as cardiac failure symptoms or an incidental finding of a cardiac murmur. Some patients are asymptomatic or have minimal symptoms; therefore, they are not diagnosed until adulthood [7, 8]. The patient we reported on in the present study is one such case. AVT may occur as an isolated lesion or associated with other cardiac diseases. AV lesions are most frequent in association, ranging from bicuspid valves without obstruction to dysplasia and even atresia. However, their association with AV aneurysm and MV chordal rupture has not yet been reported. AV aneurysm is sporadic and often associated with infective endocarditis [9]. The common causes of MV chordal rupture are degeneration and infective endocarditis. There was no typical evidence, such as fever, positive blood culture, and vegetation, to support infective endocarditis in this case. However, it was still not possible to rule out that the AV and MV lesions resulted from infective endocarditis. In addition, long-term turbulence caused by AVT could also result in an AV aneurysm, and degeneration may cause the MV chordal rupture.

The differential diagnosis of the AVT includes ventricular septal defect and rupture of the sinus of Valsalva. In the present study, we did not consider the ventricular septal defect because: (1) the flow shunt was not directly from the LV to the RV; (2) the spectrum was not only in the systolic period. The ruptured sinus of Valsalva was also not considered because (1) the ruptured sinus of Valsalva mainly occurs in aneurysmal dilated sinuses, which did not happen in our case; (2) there is no tunnel in the ruptured sinus of Valsalva, which did exist in our case.

Once the diagnosis of AVT is established, AVT closure should be performed as soon as possible. Although there are case reports of percutaneous device closure of the defect [10, 11], surgical correction is considered the optimal and standard treatment strategy. Moreover, the patch closure of each orifice is recommended [12, 13]. Our case received surgical closure for three openings of the AVT. Associated lesions of the AV and MV are treated on an individualized basis. Our case received AV and MV replacements. AV replacement was chosen instead of AV repair as the AV calcification was with a small aneurysm. MV replacement was selected instead of MV repair because (1) a significantly enlarged LV can increase the failure risk of MV repair, and (2) the patient was elderly. If the MV repair failed, he might not have endured the re-operation.

In conclusion, AVT is a rare congenital heart disease that commonly manifests as an abnormal communication between the ascending aorta and one of the ventricles. Herein, we presented a unique AVT case with abnormal communication among the ascending aorta, LV and RV. This special AVT has three orifices, i.e., one aortic opening, one LV opening, and one RV opening. This paper described the entire process from diagnosis to treatment of this unique case, thus providing some novel insights into AVT.

## Supplementary Information

**Additional file 1. **

**Additional file 2. **

**Additional file 3. **

**Additional file 4. **

**Additional file 5. **

**Additional file 6. **

**Additional file 7. **

**Additional file 8: Table S1.** The main parameters of the laboratory test.

## Data Availability

Records and data pertaining to this case are in the patient's secure medical records in the Second Affiliated Hospital of Nanchang University.

## Abbreviations

AVT: Aorto-ventricular tunnel
ALVT: Aorto-left ventricular tunnel
ARVT: Aorto-right ventricular tunnel
AV: Aortic valve
MV: Mitral valve
LV: Left ventricle
RV: Right ventricle
LA: Left atrium
RA: Right atrium
ECG: Electrocardiogram
TTE: Transthoracic echocardiography
CDFI: Color Doppler flow imaging
CTA: Computed tomography angiography
CAG: Coronary artery angiography
