# Supplementary material for: Aorto-ventricular tunnel with three orifices: a unique case report diagnosed by transthoracic echocardiography
Source: Cardiovasc Ultrasound. 2023 Apr 1;21:5. doi: 10.1186/s12947-023-00303-x (PMC10067305; doi:10.1186/s12947-023-00303-x)
Supplement: Supplementary file 8 — Additional file 8: Table S1. The main parameters of the laboratory test. [file 12947_2023_303_MOESM8_ESM.docx]

**Table S1. The main parameters of the laboratory test.**

| **Items** | **Parameters** | **Abbreviation** | **Values** | **Unit** | **Reference range** |
| --- | --- | --- | --- | --- | --- |
| Autoimmune markers | Anti-double-stranded DNA antibody | Anti-ds DNA | Negative | / | Negative |
|  | Antinuclear antibody | ANA | Negative | / | Negative |
|  | Anti-centromere antibody | ACA | Negative | / | Negative |
|  | Anti-cytoplasmic antibody | ANCA | Negative | / | Negative |
|  | Anti-complement C1q antibody | Anti-C1q | 2.96 | U/mL | 0～10 |
|  | Anti-Smith Antigen Antibody | Anti-Sm | 0.14 | AI | 0~1 |
|  | Anti-ribonucleoprotein antibody | Anti-RNP | 0.21 | AI | 0~1 |
|  | Anti-Sjogren's Syndrome A 60 | Anti-SSA-60 | 0.08 | AI | 0~1 |
|  | Anti-Sjogren's Syndrome A 52 | Anti-SSA-52 | 0.05 | AI | 0~1 |
|  | Anti-Sjogren's Syndrome B | Anti-SSB | 0.07 | AI | 0~1 |
|  | Anti-topoisomerase I-70 antibody | Anti-Scl-70 | 0.08 | AI | 0~1 |
|  | Anti-histinyl-tRNA synthetase antibody | Anti-Jo1 | 0.12 | AI | 0~1 |
|  | Anti-centromere B antibody | Anti-CENP-B | 0.03 | AI | 0~1 |
|  | Anti-chromatin antibody | Anti-CHR | 0.22 | AI | 0~1 |
|  | Anti-histone antibody | AHA | 0.31 | AI | 0~1 |
|  | Anti-ribonucleoprotein antibody | Anti-RNP | 0.03 | AI | 0~1 |
|  | Anti-mitochondrial M2 antibody | AMA-M2 | 0.05 | AI | 0~1 |
|  | Anti-PM/Scl antibodies | Anti-PM/Scl | 0.02 | AI | 0~1 |
|  | Perinuclear anti-neutrophil cytoplasmic antibody | P-ANCA | Negative | / | Negative |
|  | Cytoplasmic anti-neutrophil cytoplasmic antibody | C-ANCA | Negative | / | Negative |
|  | Atypical anti-neutrophil cytoplasmic antibody | A-ANCA | Negative | / | Negative |
|  | Anti-myeloperoxidase antibody | MPO-ANCA | 4.7 | AU/mL | 0~20 |
|  | Anti-protease 3 antibody | PR3-ANCA | 2.1 | AU/mL | 0~20 |
| Inflammatory markers | Anti-cyclic citrulline polypeptide antibody | Anti-CCP | <0.50 | U/mL | 0-5 |
|  | Antistreptolysin "O" | ASO | 48.9 | IU/mL | 0~116 |
|  | Rheumatoid factor | RHF | <20.0 | IU/mL | 0~20 |
|  | Serum C-reactive protein | CRP | 2.88 | mg/L | 0~8 |
|  | Erythrocyte sedimentation rate | ESR | 2 | mm/h | 0~15 |
| Cardiac marker | N-terminal pro-brain natriuretic peptide | NT-pro-BNP | 641 | pg | 0~300 |
|  | Creatine kinase | CK | 73.6 | U/L | 50~310 |
|  | Creatine kinase MB isoenzyme | CK-MB | 15.8 | U/L | 0~24 |
|  | Lactate dehydrogenase | LDH | 248.3 | U/L | 120~250 |
|  | Ultra-sensitivity troponin I | TnI-Ultra | 0.02 | ng/ml | 0～0.04 |
| Complete blood count | White blood cell count | WBC | 4.31 | 109/L | 3.5~9.5 |
|  | Red blood cell count | RBC | 4.32 | 109/L | 4.3~5.8 |
|  | Hemoglobin | HGB | 131 | g/L | 130～175 |
|  | Platelet | PLT | 165 | 109/L | 120～350 |
| Hepatic function | Total bilirubin | TBIL | 18.5 | umol/L | 0~25 |
|  | Direct bilirubin | DBIL | 5.3 | umol/L | 0~6.84 |
|  | Indirect bilirubin | IBIL | 13.2 | umol/L | 0~18.16 |
|  | Aspartate aminotransferase | AST | 35.3 | U/L | 15~40 |
|  | Alanine aminotransferase | ALT | 46.1 | U/L | 9~50 |
| Renal function | Urea | UREA | 7.8 | umol/L | 3.6~9.5 |
|  | Creatinine | Cre | 101.9 | umol/L | 57~111 |
| Coagulation function | Fibrinogen | FIB | 2.1 | g/L | 2~4 |
|  | Prothrombin time | PT | 12.6 | sec | 9~13 |
|  | International normalised ratio | INR | 1.1 |  | 0.8~1.2 |
|  | Thrombin time | TT | 16.1 | sec | 14~24 |
|  | Activated partial thromboplastin time | APTT | 30.3 | sec | 20~40 |
| Serum electrolytes | Potassium | K | 4.37 | mmol/L | 3.5~5.3 |
|  | Sodium | NA | 146.8 | mmol/L | 137~147 |
|  | Chlorine | CL | 101.7 | mmol/L | 99~110 |
|  | Calcium | Ca | 2.4 | mmol/L | 2.11~2.52 |
| blood glucose and lipids | Glucose | GLU | 4.67 | mmol/L | 3.9~6.1 |
|  | Cholesterol | CHOL | 4.21 | mmol/L | <5.18 |
|  | Triglyceride | TG | 1.23 | mmol/L | <1.7 |
